# Reported Outcome Measures in Degenerative Cervical Myelopathy: A Systematic Review

**DOI:** 10.1371/journal.pone.0157263

**Published:** 2016-08-02

**Authors:** Benjamin M. Davies, Maire McHugh, Ali Elgheriani, Angelos G. Kolias, Lindsay A. Tetreault, Peter J. A. Hutchinson, Michael G. Fehlings, Mark R. N. Kotter

**Affiliations:** 1 Academic Neurosurgery Unit, Department of Clinical Neurosurgery, University of Cambridge, Cambridge, United Kingdom; 2 WT MRC Cambridge Stem Cell Institute, Anne McLaren Laboratory, University of Cambridge, Cambridge, United Kingdom; 3 Toronto Western Hospital, University Health Network & University of Toronto, Toronto, Canada; 4 John van Geest Brain Repair Centre, University of Cambridge, Cambridge, United Kingdom; Emory University School of Medicine, UNITED STATES

## Abstract

**Objective:**

Degenerative cervical myelopathy [DCM] is a disabling and increasingly prevalent group of diseases. Heterogeneous reporting of trial outcomes limits effective inter-study comparison and optimisation of treatment. This is recognised in many fields of healthcare research. The present study aims to assess the heterogeneity of outcome reporting in DCM as the premise for the development of a standardised reporting set.

**Methods:**

A systematic review of MEDLINE and EMBASE databases, registered with PROSPERO (CRD42015025497) was conducted in accordance with PRISMA guidelines. Full text articles in English, with >50 patients (prospective) or >200 patients (retrospective), reporting outcomes of DCM were eligible.

**Results:**

108 studies, assessing 23,876 patients, conducted world-wide, were identified. Reported outcome themes included function (reported by 97, 90% of studies), complications (reported by 56, 52% of studies), quality of life (reported by 31, 29% of studies), pain (reported by 29, 27% of studies) and imaging (reported by 59, 55% of studies). Only 7 (6%) studies considered all of domains in a single publication. All domains showed variability in reporting.

**Conclusions:**

Significant heterogeneity exists in the reporting of outcomes in DCM. The development of a consensus minimum dataset will facilitate future research synthesis.

## Introduction

Chronic compression of the cervical spinal cord can arise from a range of disease processes, including spondylosis, stenosis, disc herniation, ligament hypertrophy or ossification. Collectively these disorders are referred to by the encompassing term of degenerative cervical myelopathy [DCM]. [1] Symptoms often start with mild pain, loss of digital dexterity and subtle gait disturbances but typically progress, with tetraplegia a potential extreme. DCM is estimated to be the most common cause of spinal cord dysfunction worldwide. [2] In an Asian population 4 in 100,000 underwent surgery for DCM annually. [3] Given its prevalence in the elderly and with an aging population, its incidence is predicted to rise. [4]

Surgical decompression, to alleviate cord compression and prevent deficit progression, is the mainstay of current treatment. However, many controversies persist, including the type and timing of surgery, leading to wide ranging variation in practice. [5]

The emergence of an optimum treatment strategy has been complicated by the heterogeneous outcome measures used across the globe, introducing publication bias, hampering inter-study comparison and guideline creation. [6,7] This is a recognised challenge in many clinical fields and has lead to the development of minimum data sets, which consist of agreed, standardised set of data elements that should be measured and reported in a specific field of healthcare. [8]

Various methods have been developed to generate such data sets. A first step is often a systematic review, to identify the range of outcomes used in the literature. The list of reported outcomes is subsequently refined using a structured consensus process including all relevant parties; clinicians, academics, allied care professionals, patients and carers.[9,10] The latter stakeholders are key to ensuring outcomes are patient centred.[11] Organisations such as the COMET [Core outcome measures in effectiveness trials] initiative have been setup to facilitate the process. [12]

The objectives of this study were therefore to describe the range of outcome measures, and the manner in which they are reported, in studies of DCM, to inform a subsequent consensus study. The current work complements and extends a recent narrative review of ancillary outcome measures in DCM. [13]

## Method

The systematic review was conducted in accordance with the PRISMA guidelines (S1 Table) and registered with the PROSPERO prospective register of systematic reviews (CRD42015025497).[14] MEDLINE [Ovid] and Embase [Ovid] databases, from 1^st^ January 1995 to 12^th^ August 2015 were searched using the search strategy [‘Cervical’] AND [‘Myelopathy’] for articles considering myelopathy secondary to subacute compression. Animal studies, case reports and letters/editorials were excluded.

Titles and abstracts were screened for relevance, with subsequent full text articles sought and screened for eligibility according to the following criteria;

- English, full text
- Prospective study with >50 patients or retrospective study with >200 patients
- Assessment of clinical outcomes in response to a treatment stratagem (conservative or interventional)

Articles were screened by two authors [BMD, AE] and data were extracted independently by two authors [BMD, MM] using a piloted proforma. Discrepancies were settled by discussion and mutual agreement. (S2 Table, S3 Table)

Descriptive statistics were used to report frequency and proportion of outcome measures. When considering the reporting method of a single instrument, proportions were presented as the percentage of studies, which had used that instrument.

## Results

**Fig 1:**
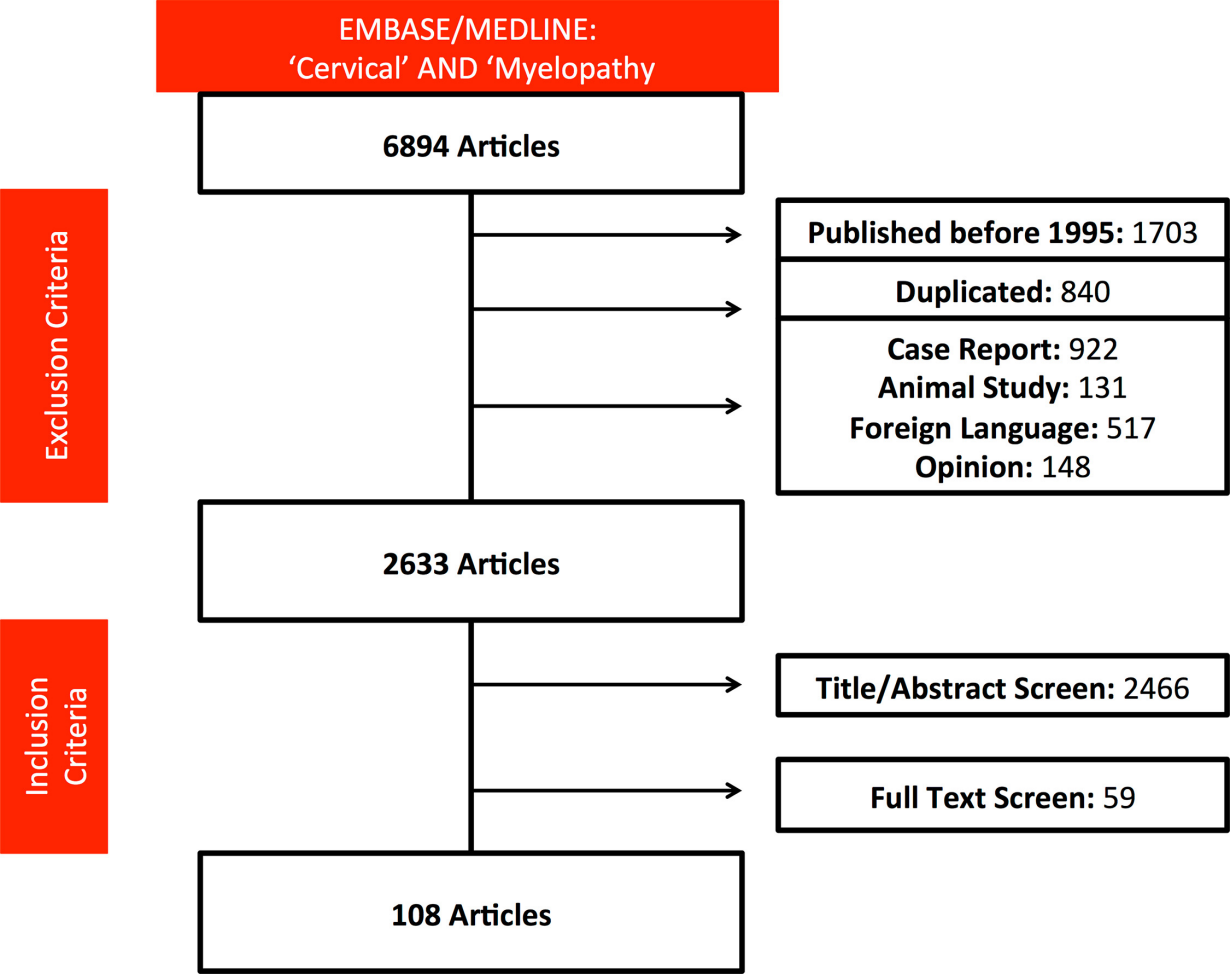
PRISMA flow diagram of the search strategy.

**Fig 2:**
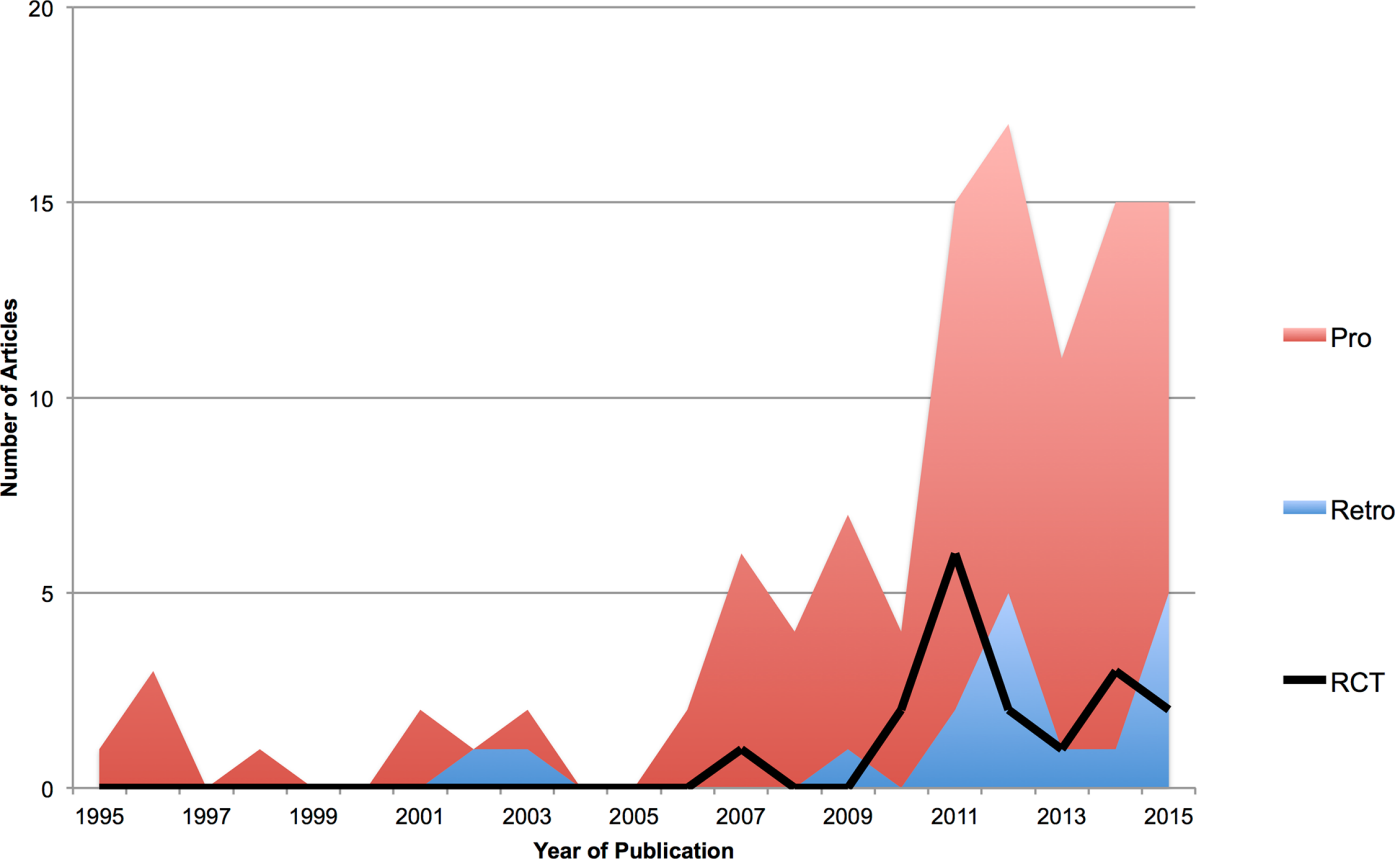
Trends in published research. Stacked line graph of year of publication of identified studies. Retrospective studies are in blue, prospective studies in red and the number of RCTs indicated by the black line. [Pro: Prospective, Retro: Retrospective, RCT: Randomised Controlled Trial]. The number of publications has increased over time, including those deemed of higher quality (prospective and randomised).

Of the 6894 articles identified, 4261 articles were excluded based on our criteria. Following abstract and title review, 170 articles were shortlisted (S2 Table) and their full published manuscripts reviewed. Of these, 108 were included in this study (S3 Table), assessing 23, 876 patients [Fig 1]. The majority of studies were conducted in North America (33, 31%), Japan (28, 26%) or other parts of Asia (36, 33%). Seventeen (16%) studies were randomised controlled trials and 91 (85%) were conducted prospectively. Publication rate, including the proportion of prospective studies and randomised controlled trials increased over time [Fig 2]. 105 [97%] studies considered patients undergoing surgery, including one study assessing CT guided treatment of disc herniation. The remaining studies considered conservative management.

Identified outcomes could be categorised into five common domains; function (reported by 97, 90% of studies), complications (reported by 56, 52% of studies), quality of life [QOL] (reported by 31, 29% of studies), pain (reported by 29, 27% of studies) and imaging (reported by 59, 55% of studies) outcomes (Table 1). Seventy-sex (70%) of studies considered more than one domain. Only 7 (7%) studies considered all of domains in a single publication, 6 of which were RCTs.

**Table 1:** Outcome domains identified by this systematic review.

Outcome Domain	Number of studies including outcome domain (%)
Function	97 (90%)
Complications	56 (52%)
Quality of Life	31 (29%)
Pain	29 (27%)
Imaging	59 (55%)
***All***	**7(7%)**

### Function

**Fig 3:**
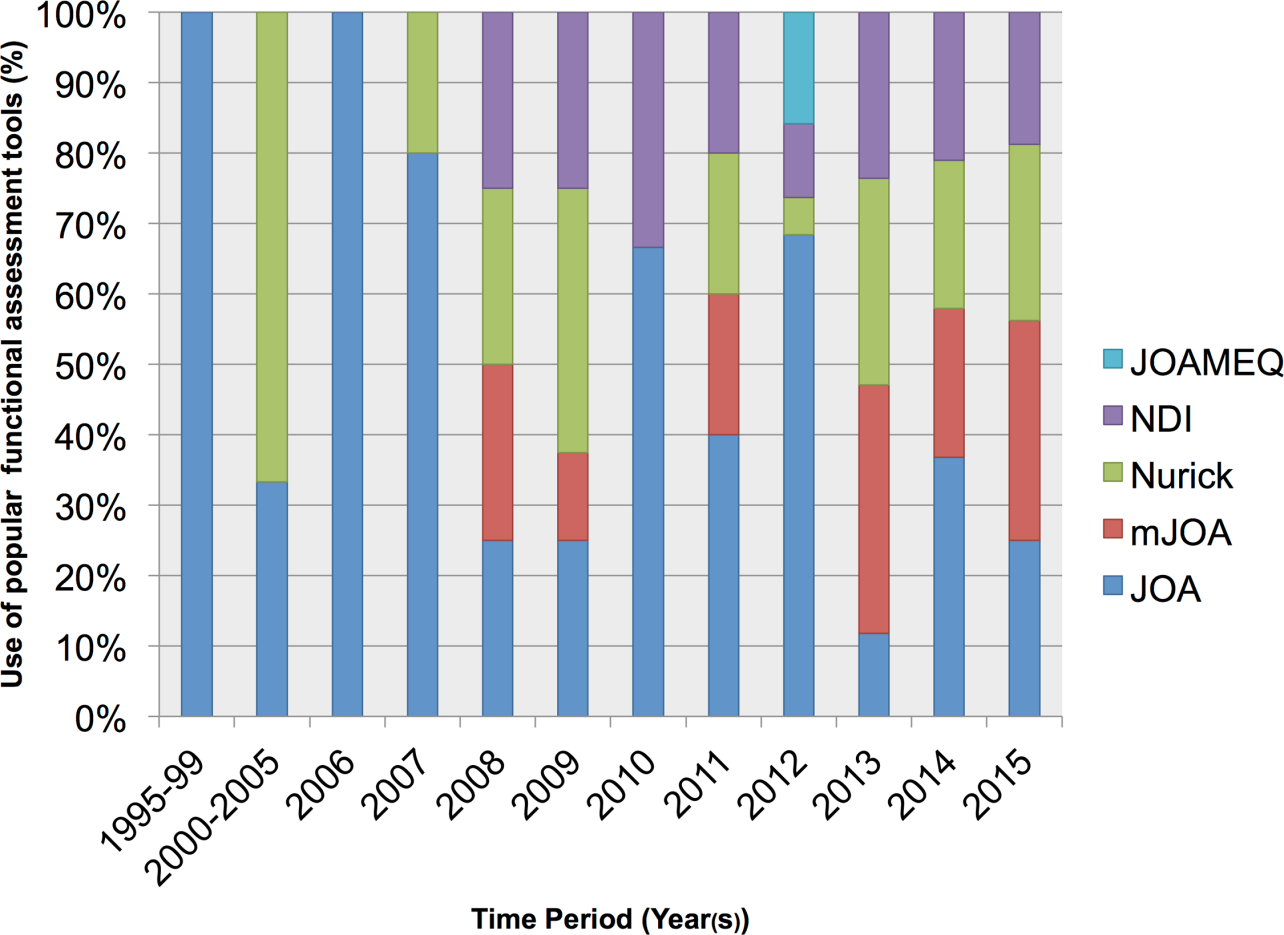
Variation in chosen functional outcomes overtime. Each bar represents 100%. The use of the most prevalent outcome measures (JOA, Nurick, NDI, mJOA, JOACMEQ) are reported as percentages for each time period.

Function was clinically assessed in 97 (90%) of studies, of which 35 (32%) used more than one tool. The Japanese Orthopaedic Association assessment [JOA], functional and neurological assessments, was most prevalent (50, 46%). Common alternatives included the gait and mobility-centric Nurick score (25, 23%), modified JOA [mJOA], an adaption of the JOA for the non-Asian population (20, 19%), and the patient-reported Oswestry Neck Disability Index [NDI] scales (20, 19%). The new JOA cervical myelopathy evaluation questionnaire, which in essence combines the mJOA with the SF-36, was used in 3 (3%) studies. The popularity of these grading systems changed over time (Fig 3) with the use of the JOA declining, in favour of mJOA and Nurick.

**Fig 4:**
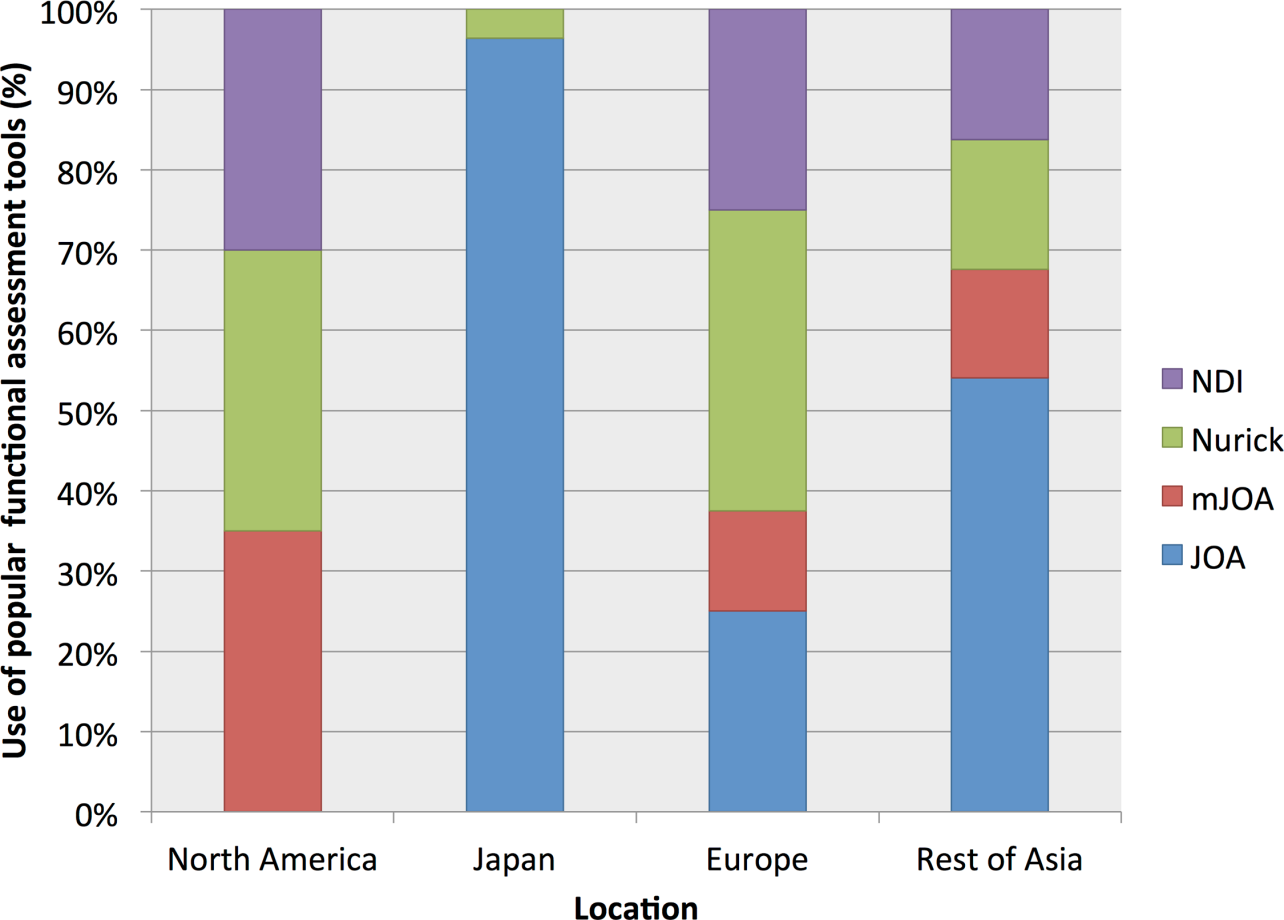
Geographical variation in chosen functional outcomes. Each bar represents 100%. The use of the most prevalent outcome measures (JOA, Nurick, NDI, mJOA, JOACMEQ) are reported as percentages for each territory.

Geographical variation was noted in the choice of functional assessment (Fig 4). For example Japanese studies predominantly used the JOA assessment, whereas this was never used in North American studies.

Other functional outcome assessments included Odom’s Criteria (8, 7%), “Neurological Success” dichotomised to the same/better or worse from examination findings (6, 6%), return to work (3, 3%) and the patient-reported Myelopathy Disability Index (2, 2%). Grip and release assessment, 30m walking test, Neurosurgical Cervical Spine Score, Mean Locomotion Score, Grip Strength, Neck range of movement and the Ranawat classification of disease severity featured once only.

The JOA was mostly reported as an overall mean (42, 84%) with or without a standard deviation (38, 76%). Most, additionally reported the JOA recovery rate (34, 68%), first described by Hirabayashi et al (1981). [15] The recovery rate was reported uniquely in two cases. Alternatives include mean difference and categorised reporting. Components of the JOA were also reported and sub-analysed in four studies (8%).

The method of reporting the Nurick grade had greater variability. Half reported a mean (13, 52%), often with standard deviation or 95% confidence intervals. Others reported subcategories, such as proportion of patients with improvement (9, 36%). The Nurick recovery rate was used twice.

The mJOA was generally reported as a mean (15, 75%), with or without standard deviation (8, 40%). In addition values were categorised (8, 40%), presented as mean difference (2, 10%) or using a recovery rate formula (1, 5%). On three occasions, the postoperative mJOA was only reported as a categorised value.

The NDI was generally reported as a mean (15, 75%), with or without standard deviation (10, 50%). Alternatives were mean difference (6, 30%) or proportion achieving a predefined improvement (3, 15%).

### Complications

56 (52%) of studies reported intervention complications. This could simply be a description of their overall frequency or a specific breakdown. Reported specific complications included C5 palsy, dysphagia, dysphonia, dural tear, CSF leak, surgical site infection and haematoma. Mortality, even if absent, was infrequently reported (15, 14%). The requirement for revision surgery, either immediate or delayed, was reported in 27 (25%) studies.

### Quality of Life

QOL was reported by 31 (29%) of studies. The Medical Outcome Short Form Health Survey [SF-36] was the predominant QOL measure used (25, 23%). The more recently developed, Japanese Orthopaedic Association Cervical Myelopathy Evaluation Questionnaire [JOACMEQ] was only used in 3 (3%) of studies. Other outcome measures included the EQ-5D (2, 2%) and the 12-item Short Form Health Survey [SF-12] (2, 2%).

The method of reporting the SF-36 varied; 9 (36%) calculated and reported each of the 8 component scores [vitality, physical functioning, bodily pain, general health perceptions, physical role functioning, emotional role functioning, social role functioning and mental health], whereas 9 (36%) reported the mental [MCS] and physical [PCS] component summary scores only. Other options included reporting only the PCS (3, 12%), the overall score (3, 12%) or everything (1, 1%).

### Pain

Pain was assessed in 29 (27%) of studies. With the exception of one study, it was reported via an assessment method distinct to a QOL tool. Pain was most commonly measured using the 10cm Visual Analogue Scale [VAS] (21, 19%). Alternatives included a numeric rating scale (1, 1%), Likert scale (2, 2%) or consultation questions as simple as *“Do you have any pain*, *yes or no*?*”*

In general pain assessment was focused (24, 22%); 17 (16%) considered upper limb pain, 22 (20%) neck pain and 9 (8%) axial pain. The term axial pain is ambiguous and it was rarely defined. One study considered it to include neck and shoulder pain, whereas another made a distinction between neck and ‘axial symptoms’.

The reporting of the VAS also varied. The majority (17, 81%) reported a mean and standard deviation before and after, with statistical comparison by a T Test. This was despite distribution analysis being reported on only 4 occasions. Alternative reporting included the percentage of patients who attained a >20mm improvement (2, 12%).

### Imaging

A total of 59 (55%) of studies reported radiological outcomes. Assessments were predominantly made using X-rays (46, 43%). CT (23, 21%) and MRI (19, 18%) were used less commonly. Radiological outcomes largely concerned fusion (29, 49%), range of movement (25, 42%), cervical alignment (25, 42%) and decompression (15, 25%). The exact definition of fusion and assessment of range of movement was variable. Cervical alignment was largely assessed using Cobb’s method (14, 56%) or the Ishihara’s Cervical Curvature Index (7, 28%). Decompression generally referred to adequate cord decompression assessed by MR (8, 53%). Alternative metrics pertain to changes in canal size.

Additional outcome measures included adjacent segment degeneration (3, 5%) or novel MR cord metrics pertaining to cord signal intensity, cross sectional area and ‘drift back’ (4, 4%).

### Other

Some studies reported treatment characteristics including length of operation (23, 21%), blood loss (22, 20%) and length of hospital (8, 7%). Cost of Care was reported twice (2%).

### Randomised Controlled Trials

Overall there were 17 RCTs, and the mean number of domains they reported was significantly greater than other studies (3.9 vs. 2.3) (Table 2).

**Table 2:** Outcome domains reported by Randomised Controlled Trials.

Article		Function	Complications	QOL	Pain	Imaging
Ying et al	2007	JOA	Reported			Fusion
						Alignment
						Other
Riew et al	2008	Nurick	Reported	SF-36	VAS	
		NDI				
*Goffin et al	2010	NDI	Reported	SF-36	SF36 Pain Score	Alignment
						ROM
Lian et al	2010	JOA	Reported		VAS	Alignment
						Fusion
*Park et al	2011					Other
Wan et al	2011	JOA			NRS	Alignment
						Fusion
						ROM
Sun et al	2011	JOA	Reported			Fusion
						Other
Sasso et al	2011	NDI	Reported	SF-36	VAS	Alignment
Cheng et al	2011	JOA	Reported	SF-36		ROM
		NDI				
Zhang et al	2012	NDI	Reported		VAS	ROM
Wang et al	2012	JOA	Reported	SF-36	VAS	Alignment
						Other
Phillips et al	2013	Nurick	Reported	SF-36	VAS	ROM
		NDI				Fusion
Nakashima et al	2014	JOA	Reported			Alignment
						ROM
						Other
Mashadin et al	2014	Nurick	Reported			Fusion
						Other
Xie et al	2015	JOA	Reported		VAS	Fusion
						Alignment
						Other
Phillips et al	2015	Nurick	Reported	SF-36	VAS	ROM
		NDI				Other
Jeyamohan et al	2015	mJOA	Reported	SF-12	VAS	Fusion

*Indicates substudy based on RCT data.

VAS Visual Analogue Scale, NDI Oswestry Neck Disability Index, JOA Japanese Orthopaedic Association, NRS Numeric Rating Scale, ROM Range of Movement.

A functional outcome was reported by all RCTs. Four RCTs reported more than one function assessment, and chosen assessments were typically the JOA (8, 47%) or NDI (7, 41%). Complications were reported by 15 (88%) and QOL by 8 (47%) of studies. The favoured QOL measure was the SF-36, used by 7 of these. Pain was assessed by 11 (65%) of studies, typically using the VAS (9, 82%). Radiographic outcomes were reported by 16 (94%) of RCTs

## Discussion

This systematic review has identified that DCM is a topic of world-wide interest, with research participation from all corners of the globe. However, there is great variation in the types of outcomes assessed and how they are reported. Key outcome domains were function (most commonly reported using the JOA), QOL (most commonly reported using the SF-36), treatment complications, pain (most commonly reported using the 10cm VAS) and imaging. Very few studies considered them all. RCTs reported more domains than other types of study and typically, when considering a domain, were more consistent in their choice of reporting.

This heterogeneity is not surprising, a survey by Singh et al (2005) of clinicians found great variability in the assessment and grading of DCM.[16] Equally it has been well demonstrated in other fields of health care. [17–19] Heterogeneity of outcome reporting is recognised to challenge inter study comparison and likely lead to bias in the dissemination of knowledge. [20] To overcome these challenges the development of standardised reporting sets has been proposed. Similarly, the present findings of this study provide a strong basis for the development of a standardised reporting set in DCM. [7,10,17]

### Limitations

The search results demonstrate that cervical myelopathy is a feature of much published research. The search strategy excluded foreign language articles and was designed to focus on contemporary and large sample studies. The global representation of included studies suggests that the foreign language exclusion is unlikely significant. Indeed, the authors propose that assessment of 20 years of published data of large sample studies, is representative of current practice.

Beyond demonstrating heterogeneity, this study aimed to collate current reporting practice, to help inform stakeholders of a future DELPHI process. When interpreting these results, it is therefore important to recognise that only outcome measures previously used will be represented. This poses a few potential problems. *First*, more recently developed assessment tools could be underrepresented (e.g JOACMEQ).[21] *Second*, the measurement properties of these scales have not been assessed here, nor whether there is current rational for the measured outcomes. *Third*, novel areas of assessment will not be represented.

In relation to this latter aspect, it is significant that patient reported outcomes [PROMs] and preference-based outcomes, topical areas for current trial development and funding[22], were poorly represented (33, 31%). The significance of patient involvement in research has become more apparent in recent years. Chalmers et al (2014) found 85% of US health research funding was wasted and concluded one of many contributing factors was the misalignment of research objectives with patient needs.[23] Consequently PROMs are now an important aspect of trial funding applications.

The significance of preference-based outcomes in trials is largely to allow economic analysis via the derivation of metrics such as quality adjusted life years [QALY]. In practice, the gold standard preference-based tests (such as standard gamble and time trade off) are replaced by utility instruments, to infer cost utility indirectly. These utility instruments are generally quality of life measures.[24] The most commonly used scales are the EQ-5D and SF-6D.[22] Whilst the SF-36 can act as a utility instrument, typically it requires derivation of the SF-6D to do so.[25] The UK advisory body for government funded healthcare, NICE, recommends the EQ-5D for cost utility assessment, when considering treatments for approval.[26]

### Future Directions

As stated in the introduction, this systematic review is the starting point for a larger process, to define the core outcomes and common data elements in degenerative cervical myelopathy [CODE-DCM]. The study aims to identify the key data elements, to advise on how they should be reported and to identify the umbrella term by which this disease process should be referred. It is registered with the COMET initiative.[27]

The results of this process will be to allow efficient and effective inter-study comparison, to support future research and the development of an optimum treatment.

The findings of this systematic review, alongside further planned work, will be used to inform a DELPHI process, made up of key stakeholders representing patients, carers, professionals and industry.[9]

## Conclusions

Significant heterogeneity exists in the outcome reporting of studies assessing management of DCM. The development of a standardised, reporting set would support the field in the future. The findings of this study will be used as part of a larger consensus process to define the core outcomes and data elements in degenerative cervical myelopathy [CODE-DCM].

## Supporting Information

S1 Table PRISMA Checklist for Systematic Reviews. The PRISMA Checklist, including page references to the location of components in this article.
(DOC)

S2 Table Shortlisted Articles. Spreadsheet providing the initially shortlisted articles.
(XLSX)

S3 Table Included articles and Extracted Data. Spreadsheet containing the extracted data (agreed by authors BMD and MM) for all included articles.
(XLSX)
